# Supplementary material for: Utilizing mentorship education to promote a culturally responsive research training environment in the biomedical sciences
Source: PLoS One. 2024 Aug 12;19(8):e0291221. doi: 10.1371/journal.pone.0291221 (PMC11318922; doi:10.1371/journal.pone.0291221)
Supplement: S1 File — (DOCX) [file pone.0291221.s001.docx]

**Supplementary File 1. Study Measures**

Survey Items Reported in Study

- How many graduate students have you mentored up to this point in your career?
  - None
  - 1
  - 2
  - 3
  - 4
  - 5
  - 6-8
  - 9-10
  - 11 or more
- How many trainees (pre-doctoral and post-doctoral) from historically underrepresented racial/ethnic groups have you mentored up to this point in your career?
  - None
  - 1
  - 2
  - 3
  - 4
  - 5
  - 6-8
  - 9-10
  - 11 or more
- When answering these questions, consider your relationships with your graduate students. Please indicate your agreement with the following statements about your mentoring.

|  | Strongly Disagree (1) | Disagree (2) | Agree (3) | Strongly Agree (4) |
| --- | --- | --- | --- | --- |
| I tried to show interest in my mentee’s projects |  |  |  |  |
| I made my mentee feel included in the lab (or field setting) |  |  |  |  |

- Please rate the following:

|  | Poor (1) | Fair (2) | Good (3) | Excellent (4) |
| --- | --- | --- | --- | --- |
| My working relationship with my research mentees |  |  |  |  |
| The overall quality of my research mentoring relationship |  |  |  |  |

CDA Items

*Attitudes*

- Please indicate how much disagree or agree with each of the following statements:

|  | Strongly Disagree (1) | Disagree (2) | Neutral (3) | Agree (4) | Strongly Disagree (5) |
| --- | --- | --- | --- | --- | --- |
| It is important to consider the mentee’s and the mentor’s race/ethnicity in mentoring relationships |  |  |  |  |  |
| Mentoring someone with a different racial/ethnic background benefits the research (eg., exposure to new ideas) |  |  |  |  |  |
| It is important for mentors and mentees to talk together about the mentee’s racial/ethnic background |  |  |  |  |  |
| It is important for mentors and mentees to discuss how race/ethnicity impacts the mentee’s research experience |  |  |  |  |  |
| My racial/ethnic identity is relevant to my research mentoring relationships |  |  |  |  |  |
| Racial/ethnic differences between mentors and mentees enrich the research mentoring relationship |  |  |  |  |  |

*Confidence*

- How confident are you in your ability to do the following in your research mentoring relationship?

|  | Not at all confident (1) | (2) | (3) | (4) | Completely Confident (5) |
| --- | --- | --- | --- | --- | --- |
| Discuss with mentees how it feels to be a minority in science |  |  |  |  |  |
| Take advantage of opportunities to address race/ethnicity in the research mentoring relationship |  |  |  |  |  |
| Recognize aspects of the research experience (e.g., lab, field work) that may make racial/ethnic minority students feel vulnerable to confirming stereotypes |  |  |  |  |  |
| Provide opportunities for mentees to talk about their racial/ethnic identity as it relates to their research experience should the occasion arise |  |  |  |  |  |
| Notice interactions in the mentoring relationship that could be insulting or dismissive to mentees because of their race/ethnicity |  |  |  |  |  |

*Behaviors*

- Please indicate how frequently each of the following has occurred in your research mentoring relationships during the past 12 months, including any current research mentoring relationships:

|  | Never (1) | Rarely (2) | Sometimes (3) | Frequently (4) | All of the time (5) |
| --- | --- | --- | --- | --- | --- |
| I intentionally created opportunities for my mentees to bring up issues of race/ethnicity as they arose |  |  |  |  |  |
| I encourage mentees to talk about how the research relates to their own lived experience |  |  |  |  |  |
| I was willing to discuss race and ethnicity with my mentees, even if it made me uncomfortable |  |  |  |  |  |
| I reflected upon how the research experience might differ for mentees from different racial/ethnic groups |  |  |  |  |  |
| I raised the topic of race/ethnicity in my research mentoring relationships when it was relevant |  |  |  |  |  |
| I implemented specific strategies to address racial/ethnic diversity in my research mentoring relationship |  |  |  |  |  |
| I approached the topic of race/ethnicity with my mentee(s) in a respectful manner |  |  |  |  |  |

Workshop Evaluation

- Please indicate how much you disagree or agree with each of the following statements:

|  | Strongly Disagree (1) | Disagree (2) | Somewhat Disagree (3) | Somewhat Agree (4) | Agree (5) | Strongly Agree (6) |
| --- | --- | --- | --- | --- | --- | --- |
| The amount of time dedicated to the workshop was sufficient |  |  |  |  |  |  |
| The workshop content was presented in a respectful and sensitive manner |  |  |  |  |  |  |
| The speakers were knowledgeable about the workshop content |  |  |  |  |  |  |
| The content was organized and easy to follow |  |  |  |  |  |  |
| The objectives of today’s workshop were clearly defined |  |  |  |  |  |  |
| The materials and activities provided in the workshop were helpful |  |  |  |  |  |  |

- How likely is it that you will make changes in your mentoring as a result of this workshop?
  - Very Unlikely (1)
  - Unlikely (2)
  - Undecided (3)
  - Likely (4)
  - Very Likely (5)
- How likely are you to recommend this workshop to other mentors?
  - Very Unlikely (1)
  - Unlikely (2)
  - Undecided (3)
  - Likely (4)
  - Very Likely (5)

Focus Group Guides

*Focus Group #1: Expectations*

Suggested length: 30-45 minutes

Introduction: As you might know, many faculty mentors in Vanderbilt Basic Sciences have participated in Culturally Aware Mentoring training in the past few years. This is one of many efforts Vanderbilt has undertaken to improve the culture and climate at Vanderbilt with respect to diversity, equity, and inclusion (especially racial and ethnic diversity), and to improve the experiences of under-represented students at Vanderbilt. The goal of our focus groups is to gain a better understanding of your experiences with mentorship at Vanderbilt. You will be asked to participate in three focus groups.

· The first will focus on your **hopes and expectations** with respect to your doctoral program at Vanderbilt generally and the mentoring you have received more specifically.

· The second (on Thursday) will focus on your **current experiences** with mentorship.

· The third (a week from today) will focus on what you would like to see in the **future**, again focusing primarily on your mentoring relationship(s).

Questions:

· Think back to what it was like for you to come to Vanderbilt. How similar or dissimilar is Vanderbilt from your undergraduate institution? What types of things were you nervous about, if any? What types of things did you feel decently confident about?

· Prior to coming to Vanderbilt, what did you expect in terms of culture and climate with respect to diversity (especially racial and ethnic diversity)? To what extent has your experience aligned with those expectations?

· Now think about your expectations of your doctoral program before you came to Vanderbilt with respect to mentorship. What were some things you hoped to experience? In what ways were your expectations met? In what ways were they not met?

· How was your relationship with your mentor changed over time? For example, if you think about your relationship with your mentor at the beginning of your program versus now, what is different? Have those changes been positive or negative?

Closing: Thank you so much for sharing your thoughts and experiences with us today. As you know, we’ll reconvene for another focus group on Thursday at 6pm. That focus group will be similar in format to this one, but will focus more specifically on your experiences with mentorship while at Vanderbilt. At the beginning of the next focus group, we will ask you if anything occurred to you in the interim that you wish you had shared during this focus group. So if, in the next day or two, you think of something you wish you had said today, you will have the opportunity to share it at the beginning of the next focus group. You are also always welcome to email me with thoughts, comments, additions, things you want to redact, etc.

*Focus Group #2: Experience*

Questions:

· At the end of the last focus group, we told you that we would start this focus group by giving you the opportunity to add anything you wish you had said during the last focus group. Does anyone have something they want to add? Anything that occurred to you during the last 3 days as important for us to understand about your expectations about Vanderbilt?

· Talk to me generally about the mentorship you receive as a part of your doctoral program – how positive or negative has the mentorship been that you receive? Alternatively, how effective has the mentorship been that you receive?

For this next set of questions, we want you to think of your primary research mentor and your current mentoring relationship with that person—we define primary research mentor as the person who is primarily responsible for providing direction and guidance to the student researcher "mentee").

· Does your mentor seem to be willing to bring up and discuss issues of race and ethnicity with you? If so, how do those conversations go? If not, how does that affect your mentoring relationship (if at all)?

· Do you think you could raise an issue related to race/ethnicity with your mentor? Does your mentor create space for that?

· Does your mentor encourage you to connect your science to your lived experience? Or conversely, does your mentor give you the impression that your lived experience is important to your science?

· To what extent does your mentor make you feel that it is acceptable and valuable for you to bring your “whole self” to your work? Do you think that all the different parts of you are welcome at Vanderbilt? In your lab?

*Focus Group #3: Future*

Suggested length: 30-45 minutes

Introduction: Thank you all for being back with us again today. We truly enjoyed our conversation with you on Thursday and look forward to hearing what you have to say today. Thursday’s conversation focused on your experiences at Vanderbilt, especially with respect to mentoring. Today we want to talk about your hopes and dreams for your education, career development, and mentoring relationships in the future.

Questions:

· At the end of the last focus group, we told you that we would start this focus group by giving you the opportunity to add anything you wish you had said during the last focus group. Does anyone have something they want to add? Anything that occurred to you during the last 5 days as important for us to understand about your experiences at Vanderbilt?

· Now for this first question, we ask you to think of your primary research mentor. You may recall from our last session that we define primary research mentor as the person who is primarily responsible for providing direction and guidance to the student researcher "mentee").

o If you could tell your mentor (or others you interact with in a mentoring role) two or three things you’d like for them to do differently, what would it be?

· As you think about mentoring others (now or in the future) what are some of the things you want to be sure you do?

o What qualities and/or practices – on the part of the mentor and the mentee – make for a good mentoring relationship?

o How is that similar or different from the mentoring you’ve received thus far at Vanderbilt?

· As we conclude our last focus group, I’m wondering what else you want us to know about your experience at Vanderbilt?

o Anything else you want us to know about mentoring specifically?

Closing: Thank you (again!) for participating in this series of focus groups. We appreciate your generosity in sharing your time and experiences with us. We are committed to using the information you gave us to improve mentoring at Vanderbilt, as well as to developing the science and practice of mentoring more generally.
