# Supplementary material for: Utilizing mentorship education to promote a culturally responsive research training environment in the biomedical sciences
Source: PLoS One. 2024 Aug 12;19(8):e0291221. doi: 10.1371/journal.pone.0291221 (PMC11318922; doi:10.1371/journal.pone.0291221)
Supplement: Attachment — Submitted filename: review_PLOSONE_PONE-D-23-25718.docx [file pone.0291221.s002.docx]

I appreciate the opportunity to review this vital work describing an institution's efforts for diversifying the scientific workforce from the faculty training perspective. It is a significant contribution, and the comments below aim to ensure that the manuscript best represents the data to the reader in a straightforward fashion and maximize the submitted manuscript's impact.

In bold are my requests for additional materials, figures, or analyses if available and actionable.

Comments to the Authors (submitted on portal):

Summary/General Impression:

The authors of “Utilizing Mentorship Education to Promote a Culturally Responsive Research Training Environment in Biomedical Science” present a case study of work conducted over several years at Vanderbilt University. The authors use a mixed methods approach to describe the represented workshops to train faculty to support the diversification of the scientific workforce.

The program, intervention, and data descriptions are worthwhile to present. These data showed that faculty had high post-survey scores, including satisfaction and confidence in cultural diversity awareness. Qualitative data demonstrates key anecdotes, which would be essential to report associated with their data descriptions.

Surveys associated with the data have been made available and could be linked in the article or added as supplementary materials, consistent with an open data policy. The manuscript will written. However, some sections could use some clarity to improve their readability.

Of concern, the current data only partly support the claim that the intervention impacted the faculty. The qualitative data present is most compellingly presented. However, the quantitative data do not support this claim. The authors could address their stated claims by linking the presented post-survey data to the pre-survey results. The authors should describe the assessment framework they used in the analysis. The authors could remake the figures to present the data more clearly and completely describe any statistical analyses in the qualitative data section, if any. Finally, the authors could move information from the introduction to the discussion and expound on the methods to support the readability and clarity of the presented research.

Major Comments:

- The quantitative data currently do not support the major claims in this article.

The pre and post-survey must be linked, and the difference in Likert scales after the intervention must be assessed and reported with corrected *p* values. The current data do not support the discussion or abstract of the paper, which states, Line 629: “.. the education also had a positive impact on the mentors’ self-ratings of the quality of their mentoring relationships and their mentoring effectiveness.”

OR Line: 35 “...increased faculty mentors’ self-reported mentorship competencies,”

You can find this example in our previous work, Rodrigues et al., 2022, PMID:36285020, which linked Likert scales to assess an intervention - if needed.

- The data presented in the manuscript are worthwhile; however, the discussion and abstract need editing to match the current data. The current data indicates that individuals had high post-survey scores. With a linked pre-survey, the analyses or data here can address the impact of the interventions, i.e., workshops.
- If the pre-survey can’t be linked, then I would recommend not mentioning or discussing the impact of the intervention in another version of the manuscript.
- The authors addressed this primary concern in the limitations of the study. However, the discussion and abstract need to be edited to not over or miss-represent the data represented
- Significant shortening is necessary for redundant verbiage throughout the article. The manuscript needs a succinct and precise description of the workshops and strategies implemented in the introduction and expounded specificity in the article's methods, particularly addressing an assessment framework used to evaluate the program and more information about the quantitative analyses.
- The authors should place the contextualization of the previous work at Vanderbilt in the discussion section of the article

.

- **A figure** describing the intervention and strategies of assessment would be helpful, including a timeline of the different events, reviews, workshops, and focus groups.

You can find an example here in the methods section, sub-section intervention.

(<https://doi.org/10.1187/cbe.21-11-0321>). PMID: 35862583

- **A figure** (flow-chart) of the participants, i.e., the total number of faculty and those who answered the assessment (pre and post-survey), i.e., the response rate
- The introduction needs more specific details of the study design and questions the study addresses, detailed later in the methods, including an evaluation framework

.

- What particular evaluation model was used to assess the program? Through the discussion, I assume an organizational learning model for advancing inclusive science - Hurtado et al. There should be a clear description of this assessment model in the methods, such as in a new program evaluation section, and introduced in the introduction and abstract of the article.
- The quantitative data section needs to be more thoroughly explained in the methods section; for example, on Line 448, Bivariate correlations between the three CDA subscales...
- Figures 1-3 should be of better quality regarding the resolution. Interpreting these averages as presented in a whiskered box plot average is difficult. It may be beneficial to show Figures 1-3 together in a stacked bar plot with the specific questions on the survey on the Y axis, the % of respondents on the X axis, and the Likert scores labeled. If the average of each Likert scale is essential to address, then a whisker plot can be added to the same figure, just as one alternative example of presenting these data.

Example of my description:

- I would’ve liked to see the **recommendations from the trainees listed in a table** and brought up in the results section. As the discussion implies, you can compare NASEM guidelines with student recommendations if they are similar.
- **Supplementary figures should include pre and post-survey and discussion questions** for the focus groups.
- **A supplementary table can include an example of workshop materials.**
- The discussion focuses on the implications of the work and vaguely justifies the statements made. It would be helpful to bring up specific data presented throughout the results to justify the points made in the discussion and the conclusion.
- Please rewrite the abstract to reflect on the data appropriately represented and specific methods used, i.e., comment on the quantitative, qualitative, and assessment framework, statistical analyses, if any, and discussion implications. Specifically, removing mention of impact or increasing scores if a pre-survey is not linked.
- The introduction briefly clarifies the culturally “responsive mentorship education program.” You could replace “explained in a later section” with a succinct program description. The program should be straightforwardly described and understood without reading the entire manuscript.

Minor Comments:

General comment: Except for the methods section, I find phrasing such as “in this paper, described in this paper, cited in this paper” redundant and unnecessary; you could consider removing these phrases

General comment: When listing integers of 10 or under, please write these out, i.e., six instead of 6 on Line 338

General comment: Where % s are listed, please also list an n= value. Using n with percentage values will help the reader understand the denominator associated with each piece of data.

General Comment: Please be consistent with using PEERs and underrepresented-students verbiage throughout the manuscript.

General Comment: For the workshop stratification and outcomes section in the results, it may be **helpful to show a figure demonstrating when each workshop was full to establish** your point here. Otherwise, I would move specific comments on the popularity of the workshops to the discussion section.

General Comment: For the Figures:

The questions in the Likert scale should match the figure label or figure label indicated in parentheses to make the figure more accessible to the reader. For example: “Discuss with mentee how it feels to be a minority in science” (Discussing Experiences).

Line 78 “noted in the opening paragraph and in other publications,” as the introduction cites previous work and citations imply “in other publications.” This phrasing is redundant and unnecessary. I would recommend simply citing the literature after “based on the lack of racial and ethnic diversity in STEM fields.”

Line 82: It is unclear what is meant by “post-workshop.” please see an above comment and clarify whether the “responsive mentorship education program” was indeed a workshop. Explicitly describing the program.

Line 83: Remove: “Where specific authors contributed uniquely to the research design or data analyses, we note this contribution with their initials.” Please note the author contributions in the authors' contribution section and are unnecessary to highlight throughout the article.

Line 182: See the comment above about the author's contributions

Lines 154-155: “in this paper” is a redundant comment

Line 526: please check if the quote is correctly listed: “I think everyone *goes* comfortable talking about that stuff with her.” If so, use [] to add what the authors think the intended word was.

Line 590: remove “-questions we didn’t ask but should have, other things that had occurred to them along the way, etc.” etc. trivializes the rest of this phrase

Line 605-620: The last quote is rather long. Can we shorten the section while providing the contextualized information needed to illustrate the point about faculty development?
