# Supplementary material for: Utilizing mentorship education to promote a culturally responsive research training environment in the biomedical sciences
Source: PLoS One. 2024 Aug 12;19(8):e0291221. doi: 10.1371/journal.pone.0291221 (PMC11318922; doi:10.1371/journal.pone.0291221)
Supplement: Attachment — Submitted filename: PLOS One Response to Reviewers.docx [file pone.0291221.s003.docx]

Sarah V. Suiter, PhD, MS

Human & Organizational Development

Vanderbilt University

230 Appleton Place Peabody #90

Nashville, TN 37203

January 14, 2024

Dear Editors,

My co-authors and I are resubmitting our paper, “Utilizing Mentorship Education to Promote a Culturally Responsive Research Training Environment in the Biomedical Sciences”for your review and consideration for publication in *PLOS One.* We are grateful to the reviewers and appreciate their thorough and helpful feedback. We have taken this feedback seriously and responded to each point in the reviews. Below, you will find a list of the reviewer’s suggestions followed by the changes we have made. In our document uploads, we have included a “clean” version of the edited manuscript, as well as a document with track changes. Please note that we made our formatting, reference, and a few minor editorial changes to the “clean” document after we accepted track changes, so those changes are not tracked in the track changes document; our substantive edits nevertheless are.

Responses to Editors’ Requests:

- Line 224: Please ensure that the word "methods" is in the plural form, as it should be "methods."
  - We have changed Method to Methods
- In the initial mention of the results, it would be helpful to state the total number of participants and then provide the percentages.
  - Both in the tables and in the results text, we have included both percentages and numbers. Because the number total number of survey respondents is 108, n’s and percentages are very similar. We also note that some questions will have fewer than 108 respondents. These have been marked where appropriate. We leave it up to the editor’s discretion whether to keep just %’s, numbers, or both
- Please provide more information about the focus group involving doctoral students. Specifically, clarify how many participated.
  - We have clarified how many doctoral students participated in the focus groups in the section on “Data Collection and Participants”
- It is important to include a section discussing the limitations of your study and discussion of potential sources of bias.
  - Thank you for this suggestion. We included a “Limitations” section between the Discussion and Conclusion sections of our original paper. We have edited the section in our resubmission to address specific issues raised by the editor and reviewers.
- Please ensure that your manuscript meets PLOS ONE's style requirements, including those for file naming.
  - We have ensured that our manuscript meets PLOS ONE’s style requirements
- In your revised cover letter, please address whether your data are prohibited from being made public for ethics reasons or if they can be accessed in a data repository
  - De-identified quantitative data from participant surveys has been made accessible via Zenodo at [https://zenodo.org/doi/10.5281/zenodo.10372674]. Qualitative data from pre-participation surveys and from interview transcripts will not be publicly available to protect participants from potential identification.
- Please include your full ethics statement in the ‘Methods’ section of your manuscript file. In your statement, please include the full name of the IRB or ethics committee who approved or waived your study, as well as whether or not you obtained informed written or verbal consent. If consent was waived for your study, please include this information in your statement as well.
  - We have included our full ethics statement in the first paragraph of our Methods section

Reviewer #1 Comments with Responses:

- The quantitative data currently do not support the major claims in this article. The pre and post-survey must be linked, and the difference in Likert scales after the intervention must be assessed and reported with corrected *p* values. The current data do not support the discussion or abstract of the paper, which states, Line 629: “.. the education also had a positive impact on the mentors’ self-ratings of the quality of their mentoring relationships and their mentoring effectiveness.” OR Line: 35 “...increased faculty mentors’ self-reported mentorship competencies.” If the pre-survey can’t be linked, then I would recommend not mentioning or discussing the impact of the intervention in another version of the manuscript.
  - Thank you for this suggestion. We are unable to link pre- and post- tests for all participants, thus have removed any verbiage that would suggest change over time (e.g., impact, effectiveness, increased)
- Significant shortening is necessary for redundant verbiage throughout the article. The manuscript needs a succinct and precise description of the workshops and strategies implemented in the introduction and expounded specificity in the article's methods, particularly addressing an assessment framework used to evaluate the program and more information about the quantitative analyses.
  - We have shortened the article from 35 pages (excluding figures and references) to 30 pages (excluding figures and references), provided a succinct description of the workshop in the introduction, and provided more specificity on methods, including an assessment framework.
- The authors should place the contextualization of the previous work at Vanderbilt in the discussion section of the article
  - We have removed some of the contextualization text from the manuscript all together in an effort to shorten the overall length. We have retained some of the text between the Introduction and Methods section (in the “Case Study” section) because we think understanding the context of the study and what else was going on at Vanderbilt at the time of the mentoring workshops is important for helping the reader situate and interpret the findings we present.
- **A figure** (flow-chart) of the participants, i.e., the total number of faculty and those who answered the assessment (pre and post-survey), i.e., the response rate
  - As stated earlier, we are unable to link the pre- and post-surveys, and are not necessarily sure that everyone who took a post-survey took a pre-survey, as participation in the surveys was voluntary and participants were allowed to take one without the other. With this in mind, we think a figure of this type is likely to be more confusing than helpful. In the text of the manuscript, we have clarified that we only used one question from the pre-survey in our analyses/results, and the rest of the questions were from the post-survey. We have clarified response rates for the post-text surveys.
- The introduction needs more specific details of the study design and questions the study addresses, detailed later in the methods, including an evaluation framework
  - We have provided more specific details of the study design in the final paragraph of the discussion. See the response directly following this one regarding the evaluation framework.
- What particular evaluation model was used to assess the program? Through the discussion, I assume an organizational learning model for advancing inclusive science - Hurtado et al. There should be a clear description of this assessment model in the methods, such as in a new program evaluation section, and introduced in the introduction and abstract of the article.
  - Thank you for this suggestion - it helped us realize that we needed to clarify how we used Hurtado’s model. We did not initially set out to study the mentor education workshops as part of a Vanderbilt-specific case study. Rather, the case study emerged overtime, and the data we garnered informed local (department, workshop) specific actions before they were used for this case study. Because of this, reporting an evaluation model up front as if it informed data collection methods and questions is disingenuous. Rather, we used Hurtado’s model to help situate our analyses and their interpretations vis-a-vis institutional change literature. We have clarified this in the Methods section, and have moved Hurtado’s model from the Introduction to the Methods section. We have not added a new Program Evaluation section.
- The quantitative data section needs to be more thoroughly explained in the methods section; for example, on Line 448, Bivariate correlations between the three CDA subscales…
  - A sentence in the methods section has been included to state that Pearson’s bivariate correlations were calculated for the three CDA mean scale scores.
- Figures 1-3 should be of better quality regarding the resolution. Interpreting these averages as presented in a whiskered box plot average is difficult. It may be beneficial to show Figures 1-3 together in a stacked bar plot with the specific questions on the survey on the Y axis, the % of respondents on the X axis, and the Likert scores labeled. If the average of each Likert scale is essential to address, then a whisker plot can be added to the same figure, just as one alternative example of presenting these data.
  - We have taken the reviewer’s suggestions to convert the Likert scale plots to stacked bar plots, and refrain from using means and distributions and rather report n’s and percentages of respondents in the text instead.
- I would’ve liked to see the **recommendations from the trainees listed in a table** and brought up in the results section. As the discussion implies, you can compare NASEM guidelines with student recommendations if they are similar.
  - We have included recommendations from the mentees in at table (Table 4). When we edited our discussion, we removed the section in which NASEM guidelines were listed, so we have not included them in the table.
- Supplementary figures should include pre and post-survey and discussion questions for the focus groups.
  - We have included post-survey and focus group questions as supplementary materials. We have only reported on one question from the pre-participation survey, and have included that in the text. To save space and avoid redundancy, we only include questions and scales that are reported in the text in our supplementary figure
- A supplementary table can include an example of workshop materials.
  - The curriculum is quite involved and was developed based on multiple evidence-based curricula (see lines 158 - 162). Including a table with examples of workshop materials would be incredibly long and not terribly helpful to readers. We have included a link to CIMERProject.org, where a few of these curricula are available as open access.
- The discussion focuses on the implications of the work and vaguely justifies the statements made. It would be helpful to bring up specific data presented throughout the results to justify the points made in the discussion and the conclusion.
  - We have edited the discussion to ensure that the statements are supported by data presented in the results section.
- Please rewrite the abstract to reflect on the data appropriately represented and specific methods used, i.e., comment on the quantitative, qualitative, and assessment framework, statistical analyses, if any, and discussion implications. Specifically, removing mention of impact or increasing scores if a pre-survey is not linked.
  - We have updated the abstract to reflect the methods we used and have removed mention of impact
- The introduction briefly clarifies the culturally “responsive mentorship education program.” You could replace “explained in a later section” with a succinct program description. The program should be straightforwardly described and understood without reading the entire manuscript.
  - Thank you for this suggestion. We have done this.
- General comment: Except for the methods section, I find phrasing such as “in this paper, described in this paper, cited in this paper” redundant and unnecessary; you could consider removing these phrases
  - We have removed these phrases
- General comment: When listing integers of 10 or under, please write these out, i.e., six instead of 6 on Line 338
  - We have made this change
- General comment: Where % s are listed, please also list an n= value. Using n with percentage values will help the reader understand the denominator associated with each piece of data.
  - We have included n values wherever there are percentages listed
- General Comment: Please be consistent with using PEERs and underrepresented-students verbiage throughout the manuscript.
  - We have worked to ensure we were consistent with PEER throughout the manuscript.
- General Comment: For the workshop stratification and outcomes section in the results, it may be **helpful to show a figure demonstrating when each workshop was full to establish** your point here. Otherwise, I would move specific comments on the popularity of the workshops to the discussion section.
  - We have removed the comment regarding popularity of the workshops
- General Comment: For the Figures: The questions in the Likert scale should match the figure label or figure label indicated in parentheses to make the figure more accessible to the reader. For example: “Discuss with mentee how it feels to be a minority in science” (Discussing Experiences).
  - We have included matching labels for the figures in the legends
- Line 78 “noted in the opening paragraph and in other publications,” as the introduction cites previous work and citations imply “in other publications.” This phrasing is redundant and unnecessary. I would recommend simply citing the literature after “based on the lack of racial and ethnic diversity in STEM fields.”
  - We have made this change
- Line 82: It is unclear what is meant by “post-workshop.” please see an above comment and clarify whether the “responsive mentorship education program” was indeed a workshop. Explicitly describing the program.
  - We have worked to clarify our naming of the mentoring intervention throughout (it was indeed a workshop, and have edited throughout to indicate that it was)
- Line 83: Remove: “Where specific authors contributed uniquely to the research design or data analyses, we note this contribution with their initials.” Please note the author contributions in the authors' contribution section and are unnecessary to highlight throughout the article.
  - We have removed author initials throughout, with the exception of the description of qualitative analysis – in this section, we have retained initials because describing who did what is important to understanding our analytic process
- Line 182: See the comment above about the author's contributions
  - We have removed initials throughout
- Lines 154-155: “in this paper” is a redundant comment
  - We have removed this
- Line 526: please check if the quote is correctly listed: “I think everyone *goes* comfortable talking about that stuff with her.” If so, use [] to add what the authors think the intended word was.
  - Thank you - this word should be “was” - we have changed it in the manuscript
- Line 590: remove “-questions we didn’t ask but should have, other things that had occurred to them along the way, etc.” etc. trivializes the rest of this phrase
  - We have removed this
- Line 605-620: The last quote is rather long. Can we shorten the section while providing the contextualized information needed to illustrate the point about faculty development?
  - Yes - we have edited the quote to be more succinct, and broken it in to two (the same student said it, but they were presenting two different ideas - we have split the quote accordingly)

Reviewer #2 Comments with Responses:

- I think the manuscript could benefit from being reworked as a traditional manuscript with introduction, methods, results, and discussion format.
  - We have considerably shortened our Introduction and Case Study section, and our paper now follows this format, with the exception of a brief “Case Study” section after the Introduction. If the reviewers prefer, we think this section could also be named “Study Context”
- There is a lot of information in the Introduction and Case Study sections that could be used in the discussion to better put this study into context.
  - We have removed information from the Introduction and Case Study and have edited the Discussion to clarify our primary takeaways with respect to the study.
- The introduction and Mentorship Education as a Systemic Intervention sections are repetitive – the way it reads is like there are two introductions. These sections could be pared down and streamlined.
  - We have edited and integrated these two sections into one.
- I think the paragraph in lines 120 to 130 could be moved to the discussion. It’s confusing to have it presented here because you haven’t introduced anything about the data collection yet.
  - We have removed this from the introduction
- Do you have any measure of how many mentors were invited to attend these workshops or how many mentees these mentors collectively have?
  - This is difficult to address succinctly and was not something we tracked from the beginning, so we have not included it in the paper. Because different trainings targeted somewhat different subsets of faculty/mentors (see table on pages 8 & 9), the number would have been somewhat different from year to year. We believe this number was around 300 faculty in 2018, 2019, and 2021. In those years, those invited faculty would have had around 450 graduate student mentees, although this does not account for everyone in their labs, of course (who also include postdocs, undergraduate and master’s students, etc.). It is also a snapshot (how many mentees those faculty would have had at that time, not before or after the workshops).
- You state that this study has 3 aims, the third of which is to see how students perceived faculty efforts at enacting culturally responsive mentoring. However, the questions posed in the focus groups don’t directly address this. The methods indicate that the focus groups focused on participants mentoring expectations, mentoring experiences, and changes to mentoring, which don’t address changes or perceptions regarding culturally responsive mentoring.
  - We have edited the third aim to better reflect the content of the focus group questions. We did not specifically ask students about whether the observed a change in their mentors’ behaviors post-workshop because were concerned about biasing their responses, and wanted a general assessment of their experiences with mentoring. We acknowledge that the third study aim as written was misleading (unintentionally) and have provided a more accurate aim.
- Information on participants in the focus groups is split up under two different headings making it difficult to understand the full picture.
  - We have combined the “Data Collection” and “Participants” sections into one section that contains Faculty Survey and Student Focus Group subheadings. We believe presentation is clearer and brings the above information together in one section instead of being split into two – thank you for this suggestion.
- Were continuous variables normally distributed? Is that why means and standard errors were calculated?
  - We have moved away from reporting means and standard errors given the nonparametric nature of the data. Instead, we’ve instead listed % responses/ns in the appropriate sections in the text
- On line 352, the authors indicate that participants were reluctant. However, to me it seems to indicate external influence as a motivator more than reluctance.
  - We have edited this statement to indicate external influence rather than reluctance
- line 448 – what kind of bivariate correlations? This should be in methods.
  - Pearson bivariate correlations for the means scale scores of the CDA scales. We have included this in the methods
- The introduction largely focused on the importance of belonging and inclusion for PEER. However, this did not appear to be a theme of the qualitative findings. Why do you think so?
  - We have removed some of the text about belonging from the introduction and case study in our efforts to shorten and simply those sections, so there is now less discussion of belonging. However, we respectfully disagree with the reviewer that belonging did not appear in the qualitative findings - although students never outright used the term “belonging,” they nevertheless highlighted attributes of it in many of their quotes (e.g., when discussing that they appreciated having peers of color but wanted to have more faculty of color; when identifying a “good mentor” as someone who respected their goals and created opportunities within their lab for the student to highlight their unique interests, etc.)
- Line 629 states that the education had a positive impact on mentors’ self-ratings for quality of their mentorship relationships and mentoring effectiveness. However, there is no indication that this is correct in this dataset since there is no control group or pre-education assessment with which to compare. Similarly, I didn’t see a lot of findings in the qualitative analysis that indicated that mentees noticed the improved efforts of their mentors because of cultural awareness training.
  - Thank you for making these points, and we agree. We have removed any statements in the manuscript that would indicate change as a result of the workshop (impact, effective, etc.). We have also clarified why students were not asked directly about the mentors’ participation in the workshop.
- There are a number of statements in the Case Study section that could benefit from further explanation. For example, the sentence on lines 174 to 176 “Certainly, there are few faculty from PEER groups, since faculty diversity in the Vanderbilt School of Medicine is considerably lower than graduate student diversity” had me questioning how much lower. I think you answer this question later on, but why introduce it here incompletely? Also, you mention on line 178 that inclusion has lagged. How do you know that inclusion has lagged? Have you measured it?
  - In our efforts to shorten the Case Study section and manuscript as a whole, we have removed these statements.
- Minor- please spell out NASEM the first time it is used.
  - We have made this change

Reviewer #3 Comments with Responses:

I do not have specific points for the authors, but rather one global one that I think seriously detracts from the manuscript. That is, the manuscript is exceedingly long. The level of detail is way too much, which makes it difficult to read. I found myself when reading that I would skim over sections that were not informative. For example:

- The reader has to read 9 pages before the methods section.
- The text repeats what is in the tables.
- All of the items for each of the measures are included in the text.
- Many of the quotes from the qualitative portion are entirely too long and there are too many of them.
- The presentation of the qualitative data extends over 7 pages.
- The discussion section is over 4 pages long.

With such lengthy sections and the granular level of detail provided, the reader loses sight of the purpose of the manuscript and the take home results.

- We have made significant efforts to shorten the manuscript, addressing many of the points above. We hope these efforts have made the manuscript easier to read and our main findings more clear.

Thank you again for the opportunity to resubmit our paper, and for your consideration of our work.

Sincerely,

Sarah Suiter

[Sarah.V.Suiter@Vanderbilt.edu](mailto:Sarah.V.Suiter@Vanderbilt.edu)
